# Randomized clinical trial on the effects of a computerized cognitive training for pediatric patients with acquired brain injury or congenital malformation

**DOI:** 10.1038/s41598-023-41810-1

**Published:** 2023-09-04

**Authors:** Claudia Corti, Viola Oldrati, Marta Papini, Sandra Strazzer, Geraldina Poggi, Romina Romaniello, Renato Borgatti, Cosimo Urgesi, Alessandra Bardoni

**Affiliations:** 1 Scientific Institute, IRCCS E. Medea, Bosisio Parini, Lecco Italy; 2 Laboratory of Cognitive Neuroscience, Department of Languages and Literatures, Communication, Education and Society, University of Udine, Udine, Italy

**Keywords:** Psychology, Human behaviour

## Abstract

Both acquired injuries and congenital malformations often cause lifelong disabilities in children, with a significant impact on cognitive abilities. Remote computerized cognitive training (CCT) may be delivered in ecological settings to favour rehabilitation continuity. This randomized clinical trial (RCT) evaluated the efficacy of an 8-week multi-domain, home-based CCT in a sample of patients aged 11–16 years with non-progressive acquired brain injury (ABI), brain tumor (BT) and congenital brain malformation (CBM). Following a stepped-wedge research design, patients were randomized into two groups: Training-first group, which started the CCT immediately after baseline assessment and Waiting-first group, which started the CCT after a period of time comparable to that required by the training (8 weeks). Post-training and long-term (6 months) changes were assessed. Both groups improved on visual–spatial working memory after the CCT, with benefits maintained after 6 months, while no other changes in cognitive or psychological measures were found. These findings suggest that a multi-domain CCT can generate benefits in visual–spatial working memory, in accordance with data from extant literature reporting that computer games heavily engage visuo-spatial abilities. We speculate that is tapping on the same cognitive ability with a prolonged training that may generate the greatest change after a CCT.

## Introduction

Pediatric brain alterations, both acquired injuries and congenital malformations, are known to cause lifelong disabilities in children, due to cognitive, behavioral and affective difficulties persisting over time^1–14^. From a cognitive point of view, grey and white matter anomalies of the brain may cause impairments in global intelligence as well as in core cognitive domains, such as attention, memory, executive functions, processing speed and visual–spatial abilities^5,15–17^. Also, cerebellar alterations, either in acquired or congenital diseases, may affect cognition and emotions, not limiting their effects on the motor domain^18–24^.

Numerous studies indicated that cognitive rehabilitation, also when delivered remotely, may promote use-dependent brain plasticity leading to favorable cognitive outcomes^25,26^. Indeed, the usefulness of early rehabilitation to support reorganization of cognitive circuitry has been widely recognized, due to the high level of plasticity of neuroanatomical structures, including the cerebellum, at developmental age^27–30^.

Nevertheless, a high number of patients do not receive adequate and prompt help or support for their condition in many countries^31–33^, experiencing a problematic return to the everyday settings. Remote cognitive rehabilitation allows delivering treatments out of the clinical settings, thus limiting costs and accessibility problems and ultimately reaching more patients^34,35^. A number of these programs works on computerized platforms proposing game-like exercises that are more engaging than traditional rehabilitation tasks and may help sustaining children’s motivation and adherence to the training^36^. Previous studies indicated the feasibility and efficacy of computerized cognitive training (CCT) in fostering different cognitive functions in children with neurodevelopmental disorders, including brain injury^3,4,6,37–39^.

However, evidence on efficacy is still controversial, with some experimental studies and reviews reporting limited gains or even absence of benefits^40–43^. In some cases, it was suggested that effects could be detected only if outcomes were tested through activities similar to training exercises (i.e., near transfer, but no far transfer effects), suggesting learning versus specific training effects^42^. A meta-analytic study on children with acquired brain injury (ABI) published in 2019 indicated that remote CCT programs based on the repetition of exercises (drill-based training) generate effects on visual–spatial abilities but exert no effects on other cognitive functions^3^. This supported the hypothesis of limited generalization of this type of training programs, with effects restricted to the visual–spatial domain, which has been found to be frequently enhanced also by video games in general^44,45^. Previous research also indicated that drill-based CCT programs do not have effects on the cognitive-related issues that adolescents and families experience at home^3,4,46–51^.

Up to now, numerous reviews addressed the efficacy of remote CCT for children with ABI^3,41,52–54^, while limited evidence has been gathered for those with brain tumor (BT) and congenital brain malformations (CBM)^6,55^. Proceeding from these premises, the aim of this study was to evaluate the efficacy of a remote CCT delivering drill-based exercises and simultaneously stimulating various cognitive abilities (multi-domain training) in a mixed population of children with non-progressive ABI, BT and CBM.

Previously reported data on the feasibility of this CCT was highly positive, with 97% of patients showing adherence and 94.2% of training sessions completed in the recommended timeframe of 8 weeks^3^. Preliminary data on efficacy on patients with non-progressive ABI has been also reported, indicating benefits of the program for visual–spatial working memory^4^. The present research article reports results on the whole sample of the clinical trial, which included also BT and CBM.

**Figure 1:**
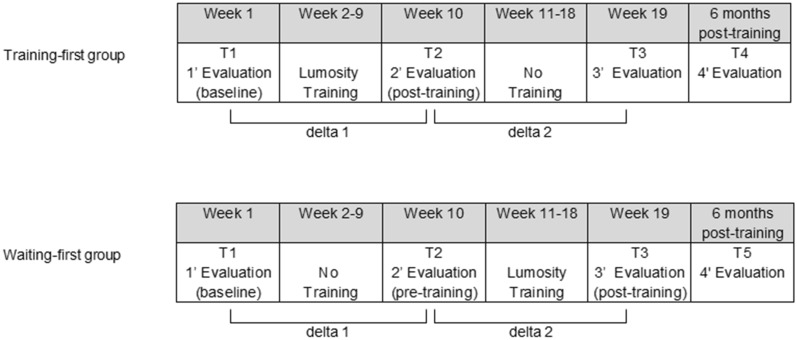
Schematic depiction of the study design.

The study utilized a randomized, stepped-wedge design (Fig. 1) where participants were divided into two groups. In the Training-first group, participants began the 8-week CCT after an initial assessment (T1), underwent a post-training evaluation (T2), and then had a waiting period before the third assessment (T3). On the other hand, participants in the Waiting-first group had an 8-week waiting-list period after the baseline assessment (T1), received a second evaluation (T2), started the training, and finally underwent the third assessment (T3), which served as the post-training evaluation. This design was implemented to isolate the effects of the training itself from any general learning effects that might have occurred during the assessment sessions. Both groups then received a fourth evaluation 6 months after the end of the training as a follow-up assessment (T4/T5). The CCT used in this study consisted of five games targeting different core cognitive abilities: memory, attention, cognitive flexibility, speed of processing, and math problem-solving. These games were selected from the available pool of exercises provided by Lumosity Cognitive Training^56^. The evaluation of training efficacy encompassed both the specific neurocognitive domains targeted by the program, using tasks distinct from those practiced in the CCT (near-transfer effects), and the assessment of psychological adjustment (far-transfer effects). In more details, the visual–spatial working memory span of the Corsi block tapping test was the primary outcome of the study. The secondary cognitive outcomes measures were: cognitive flexibility, arithmetic calculation ability, mathematical problem-solving and arithmetic speed calculation ability. As measures of psychological adjustment we used: the Child Behavioral Check List (CBCL)^57^, the Youth Self Report (YSR)^57^, the Teacher’s Form Report (TFR)^57^, the World Health Organization Quality of Life-Brief version (WHOQOL-Brief)^58^ and, finally, the Multidimensional Self-esteem Test (Test Multidimensionale dell’Autostima; TMA)^59^ for the evaluation of self-esteem. We used the following measures as covariates in the analysis: the Full-Scale Intelligence Quotient (FSIQ) and the Lumosity performance Index change (LPI-change), namely the improvement on training tasks calculated as the difference in LPI—an index of performance provided by the training—between the last and the first day of training (further details on the outcome measure and covariates can be found in the method section).

To assess each outcome measure, we computed the change between T1 and T2 (delta 1) as well as between T2 and T3 (delta 2), capturing the difference between the second and first time points. In the Training-first group, delta 1 indicates the effect of the treatment, whereas in the Waiting-first group, it reflects the spontaneous change that occurred over time without intervention. Conversely, delta 2 represents the spontaneous change observed in the Training-first group and the treatment effect in the Waiting-first group.

Delta 1 was expected to be significantly higher in the Training-first group than in the Waiting-first group, whereas delta 2 was expected to be significantly higher in the Waiting-first group than in the Training-first group. This pattern of results would indicate that the treatment effect was greater than spontaneous change in either group.

Findings of this study could help clarifying whether a drill-based CCT performed at home by patients without any clinical guidance during exercising could generate cognitive benefits, also examining whether different efficacy occurs in the three clinical subgroups. At the same time, it could inform about the usefulness of a multi-domain cognitive training in view of the fact that, up to now, no certain indication on the ideal type of cognitive training—namely single-domain or multi-domain- has been gathered. The hypothesis sustaining the choice of a multidomain training for this study was related to the consideration of the interdependence of different cognitive systems, which led us to expect that a multidomain training could lead to the greatest cognitive benefits regardless the type of brain damage^60,61^.

## Results

### Recruited participants

**Figure 2:**
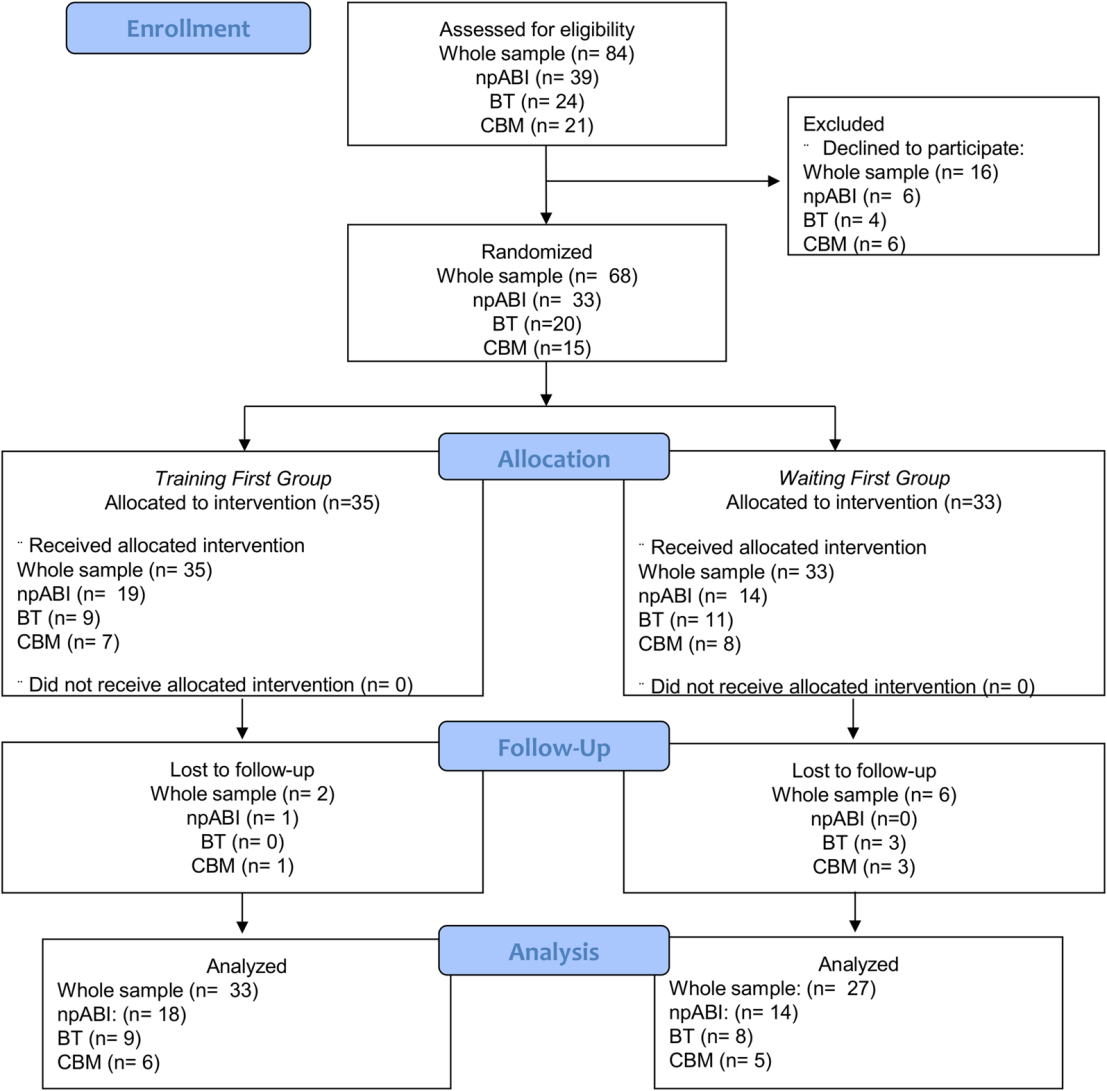
Study flowchart depicting the number of patients of each diagnostic group collected for every research step.

A total of 84 children were eligible for the study and were contacted by the research staff. Out of them, 16 children (19%) declined to participate, while the other 68 (81%) were randomly assigned to one of the two study groups. 8 children (12%) out of the enrolled 68 were lost to follow-up. Thus, the final sample comprised 60 patients, 32 with non-progressive ABI, 17 with BT and 11 patients with CBM. The Training-first group included 18 patients with non-progressive ABI, 9 with BT and 6 with CBM; the Waiting-first group included 14 patients with non-progressive ABI, 8 with BT and 5 with CBM. Study flowchart is depicted in Fig. 2.

We conducted a series of t-test to detect potential differences in demographic, cognitive and psychological adjustment variables among the Training-first group versus the Waiting-first group on the primary and secondary cognitive outcomes at T1. A significant difference was observed in the arithmetic calculation speed measure (t_58_ = 2.33, *p* < 0.05), indicating that children in the Waiting-first-group performed worse (M = − 2.47; sem = 0.43) than those in the Training-first group (M = − 1.33; sem = 0.27). All other comparisons yielded non-significant results (all *p* > 0.2). Finally, no differences between the two groups in training sessions performed was found (t_58_ = 0.71; *p* = 0.48). All data as function of treatment condition is reported in Table 1(a).

**Table 1:** Demographic characteristics and level of intellectual functioning by a) treatment condition and b) diagnostic group.

	M (SD)					
	N	sex (males)	age (in months)	FSIQ	VCI	PRI
a) Training group						
Training- first	33	21 (63.7%)	167.9 (22.4)	88.9 (19.9)	93.1 (17.7)	95.6 (21.4)
Waiting-first	27	15 (55.6%)	159.2 (25.9)	82.7 (24.2)	87.3 (21.9)	88.6 (23.0)
b) Diagnosis group						
npABI	32	23 (71.9%)	167.8 (22.5)	86.9 (23.6)	94.0 (21.4)	92.5 (22.9)
BT	17	7 (41.2%)	171.4 (23.1)	92.4 (16.2)	92.6 (15.1)	100.1 (14.9)
CBM	11	6 (54.5%)	141.5 (18.6)*	74.1 (21.6)	77.1 (16.2)*	79.1 (25)*

*Significant difference. BT, Brain tumor; CBM, Congenital brain malformation; npABI, Non-progressive ABI; FSIQ, Full scale intelligence quotient; VCI, Verbal comprehension index; PRI, Perceptual reasoning index.

The preliminary analysis, run to examine possible differences in demographical measures among the three diagnostic groups, showed a significant effect for the age of the participants (F_2,57_ = 7.12, *p* = 0.002, η^2^_p_ = 0.19), as participants in the CBM group were younger (M = 11.6; sem = 0.53) than both those in the non-progressive ABI (M = 13.7, sem = 0.31; *p* = 0.001) and those in the BT group (M = 13.9, sem = 0.43; *p* = 0.001). The distribution of female and male participants did not differ across diagnostic groups (X_2_ = 4.5, *p* = 0.1).

For what concerns the level of intellectual functioning, the analysis showed that the diagnostic groups did not differ in their FSIQ (F_2,57_ = 2.49, *p* = 0.09, η^2^_p_ = 0.08). However, they differed in the Verbal Comprehension index (VCI) (F_2,57_ = 3.39, *p* = 0.04, η^2^_p_ = 0.11), as participants in the CBM group had lower score (M = 77.1, sem = 5.72) than those both in the non-progressive ABI (M = 94.0, sem = 3.52; *p* = 0.02) and in the BT group (M = 92.6, sem = 4.59; *p* = 0.02). They also differed in the Perceptual Reasoning index (PRI) (F_2,57_ = 3.23, *p* < 0.05, η^2^_p_ = 0.10), with participants in the CBM group displaying lower scores (M = 79.1, sem = 6.46) than those in the BT group (M = 100.1, sem = 5.19; *p* = 0.01) but not as compared to those in the non-progressive ABI group (M = 92.5, sem = 3.79; *p* = 0.07). Number of sessions performed did not differ among diagnostic groups (F_2,57_ = 0.14; *p* = 0.87, η^2^ = 0.01). No participant included in the study performed less than 80% of total sessions (namely, less than 32 sessions). All data as function of diagnostic group is reported in Table 1(b).

The analysis on the psychological adjustment measures at T1 showed a significant effect of diagnostic groups for the CBCL 6–18 externalizing (F_2,57_ = 4.38, *p* = 0.02, η^2^_p_ = 0.13) and for the YSR 11–18 externalizing (F_2,57_ = 4.41, *p* = 0.02, η^2^_p_ = 0.13) scores. Indeed, the BT group had a lower CBCL 6–18 externalizing score (M = 48.1, sem = 1.91) as compared to both the non-progressive ABI (M = 54.1, sem = 1.65, *p* = 0.04) and the CBM group (M = 57.0, sem = 1.7, *p* = 0.01), whereas these last groups did not differ between each other (*p* = 0.32). In the same vein, the BT group had a lower YSR 11–18 externalizing score (M = 47.0, sem = 1.6) as compared to both the non-progressive ABI (M = 51.7, sem = 1.2; *p* = 0.05) and the CBM group (M = 54.4, sem = 2.5; *p* = 0.01), while no difference emerged between these last two groups (*p* = 0.26). No differences emerged at the other psychological adjustment measures (all *p* > 0.2).

### Primary cognitive outcome

The mixed Analyses of Variance (ANOVA) on the primary cognitive outcome, namely the visual–spatial working-memory, yielded a significant interaction of delta-time and group (F_1,58_ = 6.08, *p* = 0.02, η^2^_p_ = 0.09; main effects *p*-values > 0.2). When controlling for the possible influence of individual intellectual ability and practice-related improvements on the trained tasks, inserting FSIQ and LPI-change as covariates, the interaction delta time x group remained significant (F_1,58_ = 5.49, *p* = 0.02, η^2^_p_ = 0.09). No interaction effects were found between FSIQ and delta time (F_1,56_ = 3.04, *p* = 0.09, η^2^_p_ = 0.05) and between LPI-change and delta time (F_1,58_ = 0.82, *p* = 0.37, η^2^_p_ = 0.01).

Duncan post-hoc analyses on visual–spatial working memory revealed that, in the Training-first group, delta 1 (M = 0.64, sem = 0.19) was significantly larger than delta 2 (M =  − 0.18, sem = 0.15; *p* < 0.001), with a large effect (Cohen’s d = 0.72). This indicates that in the Training-first group performance improved more after the training (delta 1) than after a non-training condition (delta 2). In contrast, in the Waiting-first group, the difference between delta 1 (M = 0.11, sem = 0.21) and delta 2 (M = 0.37, sem = 0.17) was not significant (*p* = 0.2), although it was in the expected direction and indicating a small to medium improvement after training (Cohen’s d = 0.28). Moreover, the difference between the two groups in delta 1 was marginally significant but in the expected direction, with delta 1 being larger in the Training-first group than in the Waiting-first group (*p* < 0.07; Cohen’s d = 0.49). Similarly, the between-group difference in delta 2 was significant, being larger in the Waiting-first group than in the Training-first group (*p* = 0.02; Cohen’s d = 0.61).

For the Training-first group, the comparison between T4 (6-month follow-up after the training) and T1 (pre-training), aimed at assessing CCT long-term effects, showed a significant long-term effect of the training on visual–spatial working memory (t_32_ = 2.32, *p* = 0.03; Cohen’s d = 0.43). Similarly, the comparison between T5 (6-month follow-up) and T2 (pre-training), aimed at assessing CCT long-term effects in the Waiting-first group, showed a significant long-term effect of the training on the main outcome (t_26_ = 2.28, *p* = 0.03; Cohen’s d = 0.39).

To compare the treatment effects on the primary cognitive outcome between the three diagnostic groups (npABI, BT, CBM), we run a between-subject ANOVA on the measure of treatment effects across training groups (i.e., delta 1 for the Training-first participants and delta 2 for the Waiting first participants). The analysis showed that the improvement effect did not differ among groups (F_2,57_ = 0.75, *p* = 0.47, η^2^_p_ = 0.03).

**Figure 3:**
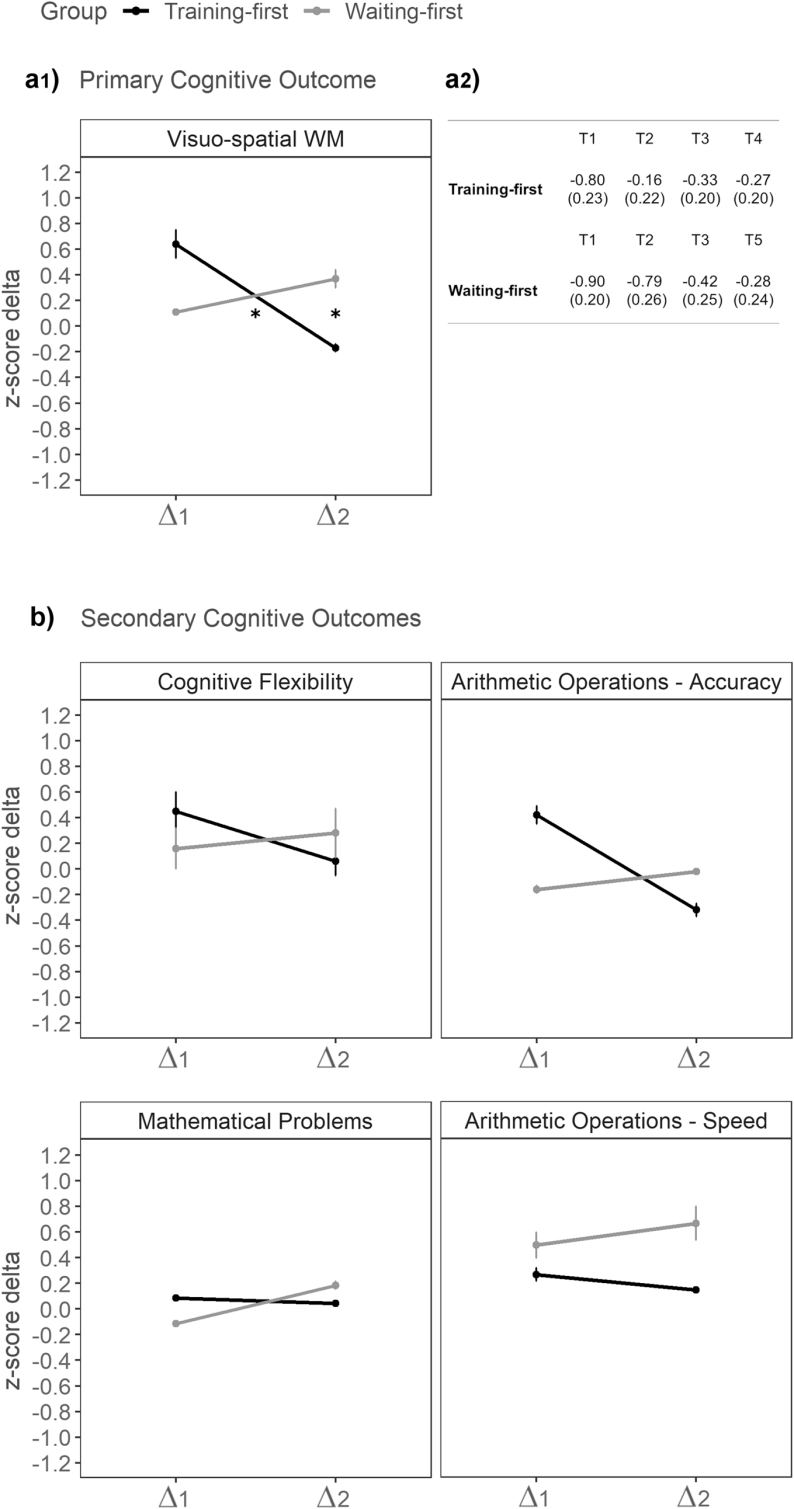
Delta change values (delta 1 and delta 2) for Training-first and Waiting-first groups in a1) the primary cognitive outcome and b) the secondary cognitive outcomes. Error bars represent ± 1 SEM. Note. Delta 1 represents the difference in performance between T1 and T2; delta 2 represents the difference in performance between T2 and T3. The right-top panel a2) reports means and SEM of the primary cognitive outcome expressed in z-scores (with values approaching 0 indicating performance closer to the normative level) across time-points and per group. For the Training-first group, the largest increase in the value was observed between T1 and T2, whereas for the Waiting-first group it was observed between T2 and T3, i.e., in both cases, after the completion of the training.

Figure 3 depicts the delta change values (delta 1 and delta 2) for Training-first and Waiting-first groups in visuo-spatial working memory and other secondary cognitive measures.

### Secondary cognitive outcomes

The ANOVAs yields non-significant main effects and interaction for the following cognitive secondary outcome measures: cognitive flexibility (delta time F_1,58_ = 0.69, *p* = 0.41, η^2^_p_ = 0.01; group F_1,58_ = 0.05, *p* = 0.82, η^2^_p_ = 0.001; interaction F_1,58_ = 2.68, *p* = 0.11, η^2^_p_ = 0.04); arithmetic calculation accuracy (delta time F_1,58_ = 1.36, *p* = 0.25, η^2^_p_ = 0.02; group F_1,58_ = 1.06, *p* = 0.31, η^2^_p_ = 0.02; interaction (F_1,58_ = 2.91, *p* = 0.09, η^2^_p_ = 0.05); mathematical problem-solving (delta time F_1,58_ = 0.77, *p* = 0.39, η^2^_p_ = 0.01; group F_1,58_ = 0.05, *p* = 0.82, η^2^_p_ = 0.001); interaction F_1,58_ = 1.32, *p* = 0.26, η^2^_p_ = 0.02). For what concerns arithmetic calculation speed, the main effect of group was significant (F_1,58_ = 4.57, *p* = 0.04, η^2^_p_ = 0.07), indicating greater improvements across time in the Waiting-first group (M = 0.58; sem = 0.13) than in the Training-first group (M = 0.19; sem = 0.12). However, nor the main effect of delta time or the interaction effect were significant (all F < 1; *p* > 0.5).

### Secondary adjustment outcomes

With respect to psychological adjustment, the mixed-model ANOVAs on CBCL 6–18 internalizing and CBCL Total Score showed non-significant main effects of delta time (F_1,58_ < 3.4, *p* > 0.1; η^2^_p_ < 0.06) and group (F_1,58_ < 1.3, *p* > 0.3; η^2^_p_ < 0.02) and non-significant interaction between delta time and group (F_1,58_ < 1.0, *p* > 0.6; η^2^_p_ < 0.01). The analysis on the CBCL 6–18 externalizing score showed a significant effect of delta time (F_1,58_ = 5.15, *p* = 0.02; η^2^_p_ = 0.08), indicating a lower values in delta 1 (M =  − 1.45, sem = 0.54) than in delta 2 (M = 0.91, sem = 0.70), but non-significant effects of group (F_1,58_ = 1.96, *p* = 0.28; η^2^_p_ = 0.02) and delta time x group interaction (F_1,58_ = 0.14, *p* = 0.71; η^2^_p_ = 0.002). As for the YSR 11–18, the analysis yielded non-significant results on all the three scores: YSR 11–18 internalizing (delta time: F_1,58_ = 1.11, *p* = 0.29; η^2^_p_ = 0.02; group: F_1,58_ = 0.02, *p* = 0.88; η^2^_p_ = 0.0004; interaction: F_1,58_ = 1.10, *p* = 0.75; η^2^_p_ = 0.002) , YSR 11–18 externalizing (delta time: F_1,58_ = 0.05, *p* = 0.82; η^2^_p_ = 0.001; group: F_1,58_ = 0.002, *p* = 0.97; η^2^_p_ = 0.00003; interaction: F_1,58_ = 0.01, *p* = 0.93; η^2^_p_ = 0.0001) and YSR 11–18 Total (delta time: F_1,58_ = 0.42, *p* = 0.52; η^2^_p_ = 0.01; group: F_1,58_ = 0.36, *p* = 0.55; η^2^_p_ = 0.01; interaction: F_1,58_ = 0.13, *p* = 0.73; η^2^_p_ = 0.002).

The TRF 6–18 externalizing score showed a significant effect of time (F_1,58_ = 4.81, *p* = 0.03; η^2^_p_ = 0.08), indicating lower values in delta 1 than in delta 2, but not of group (F_1,58_ = 0.03, *p* = 0.86; η^2^_p_ = 0.01) or their interaction (F_1,58_ = 0.66, *p* = 0.42; η^2^_p_ = 0.01). Similar pattern of results, with lower values in delta 1 than in delta 2, emerged for the TRF 6–18 internalizing score (delta time F_1,58_ = 4.96, *p* = 0.03; η^2^_p_ = 0.08; group F_1,58_ = 2.36, *p* = 0.13; η^2^_p_ = 0.04; interaction F_1,58_ = 0.83, *p* = 0.37; η^2^_p_ = 0.01) and the TRF 6–18 Total score (delta time F_1,58_ = 6.21, *p* = 0.02; η^2^_p_ = 0.09; group F_1,58_ = 0.69, *p* = 0.41; η^2^_p_ = 0.01; interaction F_1,58_ = 0.01, *p* = 0.91; η^2^_p_ = 0.0002).

Non-significant main effects or interaction were found in the ANOVAs for the WHOQOL-Brief (delta time F_1,58_ = 0.96, *p* = 0.33; η^2^_p_ = 0.02; group F_1,58_ = 1.45, *p* = 0.23; η^2^_p_ = 0.02; interaction F_1,58_ = 0.88, *p* = 0.35; η^2^_p_ = 0.02) or the TMA (delta time F_1,58_ = 1.55, *p* = 0.22; η^2^_p_ = 0.03; group F_1,58_ = 0.04, *p* = 0.84; η^2^_p_ = 0.001; interaction F_1,58_ = 0.01, *p* = 0.94; η^2^_p_ = 0.0001) scores.

Scores at the CBCL 6–18, YSR 11–18, TRF 6–18, TMA and WHOQOL on the different time points (T1, T2, T3 and T4 for the Training-first group and T1, T2, T3 and T5 for the Waiting-first group) are reported in Table 2.

**Table 2:** Means and standard deviations of standardized test scores for each psychological outcome measure of Training-first and the Waiting-first group across time-points.

	CBCL 6–18 internalizing				CBCL 6–18 externalizing				CBCL 6–18 total				YSR 11–18 internalizing				YSR 11–18 externalizing				YSR 11–18 total			
	T1	T2	T3	T4	T1	T2	T3	T4	T1	T2	T3	T4	T1	T2	T3	T4	T1	T2	T3	T4	T1	T2	T3	T4
Training-first	58.5 (1.5)	56.3 (1.7)	56.3 (1.7)	56.5 (1.7)	53.4 (1.6)	51.8 (1.4)	52.1 (1.4)	51.8 (1.4)	56.6 (1.5)	54.6 (1.5)	54.9 (1.4)	54.9 (1.5)	55.7 (1.1)	55.4 (1.0)	55.7 (1.0)	56.1 (0.9)	51.7 (1.4)	50.8 (1.5)	50.1 (1.5)	52.9 (1.2)	53.4 (1.2)	53.1 (1.3)	53 (1.3)	54.4 (1.1)

	T1	T2	T3	T5	T1	T2	T3	T5	T1	T2	T3	T5	T1	T2	T3	T5	T1	T2	T3	T5	T1	T2	T3	T5
Waiting-first	58.0 (1.5)	57.2 (1.5)	57.6 (1.6)	57.5 (1.6)	52.2 (1.8)	51.0 (1.7)	52.4 (1.5)	52.3 (1.4)	56.5 (1.4)	55.8 (1.3)	55.7 (1.3)	55.9 (1.1)	54.5 (1.2)	54.4 (1.2)	54.7 (1.0)	55.5 (1.0)	49.7 (1.3)	48.8 (1.4)	48.0 (1.3)	49.4 (1.4)	53.1 (1.0)	52.5 (1.0)	52.5 (1.0)	53.5 (0.9)

	TRF 6–18 internalizing				TRF 6–18 externalizing				TRF 6–18 total				TMA				WHOQOL							
	T1	T2	T3	T4	T1	T2	T3	T4	T1	T2	T3	T4	T1	T2	T3	T4	T1		T2		T3		T4	
Training-first	54.7 (0.9)	53.7 (0.9)	54.8 (1.0)	54.7 (1.1)	53.8 (1.2)	52.3 (1.2)	53.0 (1.3)	53.1 (1.1)	54.0 (1.1)	53.0 (1.0)	53.4 (1.2)	54.0 (1.2)	98.6 (2.0)	98.5 (1.9)	100.2 (2.0)	100.0 (1.9)	67.3 (2.4)		68.0 (2.5)		68.7 (2.6)		71.1 (2.6)	

	T1	T2	T3	T5	T1	T2	T3	T5	T1	T2	T3	T5	T1	T2	T3	T5	T1		T2		T3		T5	
Waiting-first	54.6 (1.3)	54.8 (1.2)	55.8 (1.3)	55.4 (1.1)	54.0 (1.4)	53.0 (1.3)	53.0 (1.6)	52.5 (1.3)	55.1 (1.3)	54.4 (1.3)	55.1 (1.5)	55.2 (1.2)	92.4 (2.4)	94.0 (2.3)	93.6 (2.3)	94.1 (2.0)	66.5 (3.1)		67.3 (3.9)		62.1 (4.1)		66.0 (2.5)	

## Discussion

This study aimed at assessing the efficacy of an 8-week drill-based cognitive training program performed at home by children with non-progressive ABI, BT or CBM.

Based on data of existing literature about the positive impact of computer games on visual–spatial abilities^44,45,62–65^ and of our preliminary study on the improvement in visual–spatial working memory of children with non-progressive ABI trained with the same program^4^, we expected to observe near-transfer effects on visual–spatial working-memory in the whole sample. In contrast, we had no clear expectations of training effects on the other task-related cognitive abilities (executive functioning and mathematical abilities) and on quality of life and psychological adjustment in everyday life. In fact, our preliminary study on ABI found a trend towards significance for the improvement in mathematical operations speed only and no other gain. However, this effect emerged only in one group and could represent a learning effect rather than a benefit associated with the training.

Data of the present clinical trial confirmed the positive training effects on visual–spatial working memory in the whole sample, thus also in children with BT and CBM. Importantly, the gains were maintained in the long-term, 6 months after the training, suggesting the usefulness of a relatively short telerehabilitation period in determining long lasting changes. The reason of such a benefit could be due to the consistent training of visual–spatial processes across all CCT exercises. Indeed, the various training games, albeit focusing on different cognitive domains, addressed visual–spatial competence by requiring to: detect the orientation of a stimulus in space (Disillusion, Lost in Migration); match together (Disillusion) or recognize (Tidal Treasure, Speed Match) visually presented figures that could differ in shape and color; solve arithmetic operations contained in drops that moved vertically on the computer screen and were distributed in space (Raindrops); and maintain in working memory the shapes and colors of visual stimuli (Tidal Treasure, Speed Match). This mechanistic interpretation is sustained by previous research, which reports that computer games heavily address visual–spatial working-memory. The results indicate that the benefits of games requiring mental rotation and visualization and perceptual attention on individuals’ mental health are associated with the occupation of visual–spatial working memory^44,45,62^. Previous evidence showed that visual–spatial abilities are enhanced by video- and computer-game playing, even after a limited training period^44^. In light of this evidence, one may hypothesize that a crucial role in determining the training gains may be, indeed, the dosage of the training, meant as frequency and intensity, regardless of the nature of the content to be-stimulated.

With respect to secondary outcome measures (i.e., cognitive flexibility and mathematical operations speed and accuracy), we found no benefits, except for a speed up in arithmetic calculation after the training in the Waiting-first group but not in the Training-first group. However, the Waiting-first group had lower baseline performance than the other group, suggesting that its greater change in this cognitive measure could be more reliably ascribable to its highest margin of improvement, due to specific individual characteristics, rather than to CCT effects. Overall, this study corroborates our preliminary findings on the same clinical trial indicating the absence of benefits on cognitive abilities not intensively addressed by the program^4^. This is in contrast with studies reporting gains from a multi-domain cognitive training on global cognitive performance^66–70^.

Thus, our clinical trial suggests that, in patients with different intellectual abilities and without severe motor and sensory impairments, the highest cognitive gains are reached through intensively stimulating the same cognitive function for a certain time period. Indeed, cognitive abilities different than visual–spatial skills were trained by two exercises per day, which however lasted a very limited amount of time (in many cases one minute or less). This could explain why the training failed in improving complex abilities, such as executive functions and mathematical abilities. Up to now, different studies reporting improvement effects following multi-domain CCT enrolled healthy individuals^66–70^. The present results may suggest that, in case of brain alterations, benefits may occur only after an intensive and prolonged training of specific cognitive abilities, questioning the potentialities of a multidomain training.

Regarding psychosocial adjustment, we found no significant improvement on internalizing symptoms, self-esteem related to different domains of life nor quality of life. This lack of effects may indicate that drill-based training programs fail to address the adjustment issues that children often encounter in the everyday setting. We may, thus, conclude that a rehabilitation approach focused only on training cognitive abilities through the repetition of exercises cannot be considered the optimal solution to address psychological and behavioral problems, even when an improvement in a specific cognitive domain is found. Indeed, the present evidence does not support the hypothesis that an improvement in visual–spatial working memory could lead to cascade benefits on children’s well-being in everyday life^71–74^. These conclusions are in line with numerous studies questioning the usefulness of remote training programs offering drill-based exercises to improve adjustment in ecological settings^46–51^.

In this respect, previous research suggested that, in order to generate effects on everyday functioning, more complex training types based on metacognitive abilities should be delivered in children with neurological condition^75^. In fact, we found a change over time in externalizing symptoms, with a worsening of the whole sample’s behaviours,. Even though the average score of the index was still in the normal range, we can ascribe such a negative trend to the attention paid by parents on their children’s behaviour during challenging cognitive activities, which could have caused a greater awareness of their children’s difficulties in terms of attentional, hyperactive and oppositional behaviour. In any case, it indicates no behavioural improvement.

With respect to the comparison of training efficacy across the three clinical subgroups of patients, we found no differences, with all groups showing comparable improvements in visuo-spatial working memory. This result could reflect the low statistical power associated with the limited numerosity of children with BT and CBM compared with the non-progressive ABI group, possibly limiting the detection of group differences. At the same time, our results may suggest that the feasibility and efficacy of CCT may extend across different types of brain alterations and general cognitive functioning.

This data is conflicting with previous studies suggesting that patients with CBM frequently show a complex clinical picture characterized by behavioral and physical issues together with low intellectual abilities, which may lead to limited possibilities to obtain significant gains from a remote training^6^. In addition, previous studies showed that the important difficulties of these children could even hamper the possibility of performing a remote training, suggesting that telerehabilitation may be a viable option only for a limited subgroup of children^76,77^. Considering the functional characteristics of patients with CBM in this study, the preliminary analyses confirmed a mixed pattern cognitive and behavioral issues, as they had lower verbal competence and lower initial training scores than the other diagnosis-based groups, together with a trend towards statistical significance for lower abilities in perceptual reasoning. At the same time, they exhibited more externalizing symptoms than patients with BT. Despite this, our study provided encouraging findings in terms of training adherence and efficacy also for CBM patients. This could be due to the user-friendly characteristics of the selected CCT, namely the limited daily exercise duration, the exercise difficulty adjusted to patients’ competence and the game-like format. Thus, data of this clinical trial supports training success also for those children that present a complex functioning. However, we note that patients with severe visual, auditory or motor deficits were excluded from study enrollment, according to exclusion criteria, suggesting that motor and sensory disabilities could represent the principal impediment associated with telerehabilitation. Further studies are required to provide clear evidence on this issue, possibly suggesting whether specific training formats could be more suitable for this clinical subgroup.

Limitations of this study should be acknowledged. First, the stepped-wedge design used for this clinical trial did not involve the inclusion of an active control group performing another type of training, hampering the possibility to evaluate the specific effects of training characteristics on the outcomes. This does not allow providing more defined and evidence-based indication on the optimal training format to boost cognitive abilities, in terms of therapist’s involvement or single- versus multi-domain training. Second, data on attention and processing speed, which were planned to be assessed by means of a computerized task, could not be collected for all patients due to unpredictable technical problems which prevented the use of the task in a large number of patients at some assessment points. This, of course, impeded us to enter into the analyses these core cognitive domains, limiting the detection of other abilities that may, or may not, benefit from the training. Therefore, while the use of computer-based assessment tool ensures to obtain more reliable data, it could constitute a risk in terms of data collection that should be anticipated and prevented (e.g., by allocating a higher number of economic resources to buy a new software in case of technical problems of the target one) when planning a clinical trial. Third, the training had a short duration, which could have limited the effects on the various cognitive domains and the generalizability of benefits to every-day setting adaptation. It is still object of debate how intensive, how long and how focused on a single or multiple cognitive ability a remote cognitive training should be to generate the best gains. Fourth, study randomization was non-stratified based on clinical diagnosis, which caused a non-balanced distribution of patients across clinical subgroups and could have yielded issues related to statistical power. Finally, the findings of this work are related to those games that have been specifically selected for the study and cannot be extended to the more complex format in which the training is provided in its commercial version.

## Conclusions

This randomized clinical trial demonstrated the efficacy of a drill-based cognitive training delivering exercises tapping on different cognitive domains in improving visuo-spatial working memory in a sample of children with acquired brain damage and congenital malformation. This benefit was probably due to the continuous training of visuo-spatial abilities by all training games, which suggests that the most remarkable cognitive gains are reached through the training of the same cognitive function for a prolonged time period. Notably, benefits were maintained over time and were observed for all clinical subgroups (non-progressive ABI, BT and CBM), suggesting training flexibility in adapting to different cognitive and behavioral functioning. However, no benefits on other cognitive domains were found, indicating only a limited effect of a multidomain cognitive training on the whole cognition. Thus, this study provides support to the usefulness of a prolonged training of the same cognitive ability to reach a significant change, even though future research is needed to further test this hypothesis. With respect to generalization of benefits to patients’ functioning in everyday life, our findings confirmed previous evidence about the absence of transfer effects and, thus, about the limitation of remote drill-based training programs in improving children’s quality of life and behavioral adjustment. Importantly, in contrast with previous studies reporting training adherence issues, particularly in children with CBM^76,77^, adherence in this clinical trial was very high (81% of eligible participants agreed to take part in the study) in all clinical subgroups. However, children with severe motor and sensory deficits were excluded, which could suggest that such deficits could determine the success or the failure of a remote cognitive training program and should be considered when planning a tele-rehabilitation program.

## Methods

### Study design and procedure

A stepped-wedge research design was adopted, randomly assigning patients to two groups that differed for training and assessment timing. Randomization of patient assignment was conducted according to a coin flip procedure by means of the randomization tool of Microsoft Excel: a random number was randomly associated to each patient and determined the allocation to either the Training-first group (0 to 0.49) or the Waiting-first group (0.5 to 1). Both participant enrollment and randomization were conducted by a researcher who was not part of the research team involved in testing participants. The Training-first group underwent the baseline assessment at T1 and then started the training; at T2, after training conclusion, it received the post-training evaluation; at T3 it received the 2-month follow-up evaluation. The Waiting-first group underwent the baseline assessment at T1, before starting a waiting-list period; at T2 it received the pre-training assessment and then started the training; at T3, after the completion of the training, it underwent the post-training assessment. Finally, a follow-up assessment was conducted after 6-month from T3 for both the Training-first group (T4) and the Waiting-first group (T5).

The research team was not blinded to participants’ treatment allocation, as a report containing all training information for each participant was received weekly from Lumos Lab to monitor training adherence. However, outcome assessors and participants were blinded with respect to condition assignment.

The software G Power 3 was used to estimate the sample size^78^. A final sample of 60 patients was considered adequate to detect a within-group change of moderate effect size (Cohen’s d = 0.47) with a power of 0.95 and the alfa level set at *p* < 0.05.

All procedures used for this study are in accordance with the 1964 Helsinki declaration and its later amendments and comparable ethical standards. All data were collected at Scientific Institute, IRCCS E. Medea, Bosisio Parini, Italy. This study was conducted in accordance with CONSORT guidelines for non-pharmacological interventions^79^.

The study was registered with the ISRCTN registry, as study ID ISRCTN59250807 (https://www.isrctn.com/ISRCTN59250807. Registration date: 25/10/2017) and with the Italian Ministry of Health Trial registry (number 44249 on 09/08/2016; approval: 17/11/2016).

The study was approved by the Ethic Committee of Scientific Institute, IRCCS E. Medea, Bosisio Parini, Italy (project number 284 on 01/03/2016, subsequently amended by project number 337 on 12/07/2016). Recruitment for this study started on 02/03/2016 and ended on 27/11/2019. The whole trial ended on 27/09/2020.

### Participants

To be eligible, participants had: (1) to present a brain damage, either a CBM, a non-progressive ABI (i.e., traumatic brain injury, stroke, anoxia, meningitis, encephalitis, post-surgical meningioma and acoustic neuroma) or being survivor from a BT; (2) to be aged 11–16 years; (3) to speak Italian as a primary language. Additional specific inclusion criteria were being in chronic phase (at least 1 year after the event), for children with non-progressive ABI, and having no active disease and having received no postsurgical primary adjuvant therapies in the 6 months before enrollment, for children with BT.

Exclusion criteria were: (1) severe visual, auditory or motor deficits that could interfere with training execution and outcome assessment; (2) undergoing a parallel cognitive rehabilitation treatment; (3) a diagnosis of photosensitive epilepsy, as a computer-based training may produce negative health effects. An additional specific exclusion criterion for children with non-progressive ABI and BT was the presence of a previous diagnosis of psychiatric or cognitive problems.

Recruitment was not based on a specific FSIQ range to provide data that could be generalizable to the whole population of children with non-progressive ABI, BT or CBM, who usually displays different injury severity levels and cognitive functioning also within the same clinical subgroup^80–83^. The decision not to define an arbitrary FSIQ range for participants’ inclusion was also sustained by conclusions of previous research. Indeed, on one side, it was highlighted the need to address patients with moderate and severe cognitive deficits through cognitive interventions^84^; on the other side, it was reported the efficacy of CCT in boosting neuropsychological performance of children with a neurodevelopmental disorder, even in absence of general learning difficulties or cognitive impairments^85,86^.

Eligible children were identified from the acquired and congenital brain damage registry of the Severe Acquired Brain Injury Unit, the Neuro-oncological and Neuropsychological Rehabilitation Unit and the Neuropsychiatry and Neurorehabilitation Unit of Scientific Institute, at the IRCCS E. Medea, Bosisio Parini, Lecco, Italy. These Units provide care to children with severe non-progressive ABI, BT and CBM, respectively. In about a week after identification, the referring physician in each Unit proposed the research project to children and their parents. In case of interest, a member of the research staff contacted parents by phone to provide specific information on project objectives and methodology. We obtained written informed consent by all parents of underage children who agreed to participate into the project. Children of legal age directly fulfilled the informed consent. Participants were continuously recruited across the three Units until reaching a total sample of 60 participants who performed the training, independently from the specific diagnosis (i.e., non-progressive ABI, BT, or CBM). No stratification based on diagnosis was performed.

### Intervention

Lumosity Cognitive Training™ was the CCT used for this study^56^. The program consists of a web-based platform providing game-like exercises stimulating the following cognitive domains: memory, attention, cognitive flexibility, speed and problem-solving. Five out of the numerous games included in the CCT were chosen for this study (Table 3), each one stimulating one of the target cognitive domains. Each game was delivered twice a day, for a total of 10 daily exercises. As the CCT platform was in English and not in Italian, we selected games that relied on visual–spatial information, without the need of verbal processing. Moreover, as children with heterogeneous cognitive functioning were included in the study, we selected games whose instructions were considered easy to understand and that could be performed also by children with low cognitive functioning. The CCT is able to automatically adjust games complexity to patients’ performance, which could favor motivation of both patients with low intellectual functioning, limiting frustration, and those with high intellectual functioning, favoring challenge. A clinician member of the research staff gave oral instructions for each game at the end of the pre-training evaluation session. Moreover, each participant received a leaflet containing written instructions.

**Table 3:** Description of the training games.

Name of games	Trained cognitive function(s)	Player goal/objective(s)
Disillusion	Cognitive flexibility	The child is asked to insert a form in a matrix, matching it by color or symbol with another form according to the target form orientation (horizontal or vertical). This exercise trains the skill to respond to a task modifying the rule of matching in light of contextual information (cognitive flexibility). The more forms the child is able to match, the higher is the score
Tidal treasure	Visual–spatial memory	The child is shown a beach where different objects appear: he/she has to select an object and then all objects are covered. In the subsequent screen he/she is asked to select an object that is different from the previous one and so on. Each session includes three beaches. The child fails when he/she chooses a stimulus that has been already selected. The more objects the child selects, the higher is the score
Speed match	Processing speed and spatial working memory	The child is asked to indicate as quickly as possible whether a stimulus is the same as the last one displayed, according to the symbol presented on it. As speed performance improves, the number of trials increases, augmenting difficulty level. The more correct answers are given, the higher the score
Lost in migration	Selective attention	The child is required to indicate with the correct arrow key the direction of the central bird among a bird flock. Other birds are presented with the same or different direction from the central bird. The more correct answers are given, the higher the score. This game trains selective visual–spatial attention skill
Raindrops	Arithmetic calculation	The child is asked to solve mathematical operations contained in rain-drops. He/she is requested to give an answer before the raindrop falls into the sea at the bottom of the screen. Three game possibilities within each session are presented. The more correct calculations are performed, the higher the score

The training was performed by children at home, without the direct monitoring of the clinician. A weekly phone-based contact with a clinician member of the research staff was scheduled, with the solely aim of sustaining training adherence and motivation and recording the reasons of any eventual drop-outs; no feedback on tasks execution was provided at any time. A researcher not involved in testing received, through an automatic e-mail from the Lumosity online platform, a weekly report of the scores obtained at each game by each participant. By means of this report, it was also possible to estimate the number of sessions completed by the participant.

Participants could access the program by inserting a personal email and password, which were provided to them by the research staff during the preliminary demonstration session. The average daily training duration was approximatively 20 min. A total of 40 sessions had to be performed by each participant, with a commitment of 5 times per week in a total period of 8 weeks. The intensity and duration of this version of Lumosity Cognitive Training were chosen on the basis of the characteristics of previous CCT programs for pediatric populations with brain damage or neurodevelopmental disorders^3,6,41,86^.

### Measures

Outcome measures used to test cognitive abilities at baseline evaluation, before and after the intervention and at follow-up assessments were based on tasks characterized by different settings and stimuli than the ones proposed in the CCT. This aimed to assess whether benefits from the training could not be solely associated with practice-related effects (engagement) but occurred also for different tasks than those delivered by the program (near-transfer effects on tasks different from the training). All selected outcome measures constituted well-known tools of assessment and were standardized.

#### Primary cognitive outcome

Visual–spatial working memory: the visual–spatial working memory span assessed by the Corsi block tapping test was the primary outcome measure of the study^87–89^. In fact, all CCT games required the processing of visual–spatial information, with 2 of them (Tidal Treasure and Speed Match) significantly tapping on visual–spatial memory abilities. In the Corsi block tapping test children were asked to replicate a visual–spatial sequence on spatial separated blocks glued on a wooden tablet, in the same order as the one exhibited by an examiner. Block-tapping series of increasing length were presented and 3 trials per series were provided. The memory span corresponded to the maximum length of the series in which at least 2 trials were correctly performed. Measures were converted to z scores (M = 0, SD = 1), based on age-corrected Italian normative data^87,88^.

#### Secondary cognitive outcomes

##### Cognitive flexibility

This function was tested by administering a computerized version of the Wisconsin Card Sorting Test (WCST)^90^. Children were required to generate a rule for associating cards and to modify this rule in a flexible way on the basis of a computerized feedback. The number of total errors, composed of the sum of perseverative and non-perseverative errors, was selected as the outcome measure of this test. Measures were converted to z scores (M = 0, SD = 1), based on age-corrected normative data.

##### Problem-solving abilities

An age-appropriate problem-solving task and an arithmetic calculation task included in the Italian battery AC-MT were used^91–93^. In the problem-solving task, patients were required to solve 10 written problems involving reasoning and arithmetic abilities. In the arithmetic calculation task, patients solved 4 (for middle-school children) or 8 (for high-school children) math operations with a maximum allowed time of 60 s for each operation. A conventional score of 0 on the problem-solving task was assigned if patients were not able to solve problems. For patients not able to perform the arithmetic operation tasks, a conventional score of 0 for the accuracy parameter and the maximum allowed time for solving operations were assigned. All outcome measures were converted as z scores (M = 0, SD = 1), based on age-corrected Italian normative data^94^, with higher scores indicating better performance. While the arithmetic calculation task could be considered a near-transfer outcome measure, as it involved a task similar to the Raindrops CCT game, the problem-solving task was selected as a far-transfer outcome measure, as it required more complex reasoning abilities and did not include activities similar to those provided by the CCT^91–93^.

The original research protocol planned the computerized assessment of attention and processing speed by using the indexes ‘omissions’, ‘commissions’ and ‘Hit Reaction Time' of the Conners’ Continuous Performance Test III (CPT-3)^95^. However, due to unexpected technical problems occurred with the use of the program software, we could not record the results of many participants on CPT III at post-test and/or at 2-month follow-up. Therefore, we excluded these measures from statistical analyses.

#### Secondary adjustment outcomes

##### Psychological adjustment

The internalizing, externalizing and total scores of the CBCL 6–18, YSF 11–18) and TRF 6–18 were considered as outcome measures for the far-transfer evaluation^57^. This aimed to give an overall understanding of children’s functioning in different every-day settings. The CBCL 6–18 was filled out by parents, the YSR 11–18 by the participants themselves, and the TRF 6–18 by teachers. The three questionnaires require respondents to answer items assessing psychological and adjustment problems of the children, by providing a response on a 0 (“not true”)–2(“very/often true”) Likert scale. The CBCL 6–18 and TRF 6–18 are composed of 113-items, while the YSR 11–18 by 112 items. Scores of the three questionnaires were expressed as T-scores (M = 50, SD = 10). The higher the scores, the more the problems.

##### Quality of life with respect to psychological adjustment

WHOQOL-Brief was used to assess psychological adjustment of children^58^. Specifically, the instrument is a self-report questionnaire evaluating individuals’ perception of their position in life with respect to the following domains: physical domain (pain and discomfort, energy and fatigue, sleep and rest, mobility, activities of daily living, dependence on drugs and medical aids and work capacity); psychological domain (positive feelings, thinking, learning, memory and concentration, self-esteem, bodily image and appearance, negative feelings and religion/spirituality/personal beliefs); social relationships (personal relationships, social support and sexual activity); environment (physical safety and security, home environment, financial resources, health and social care, opportunities to acquire new information and skills, participation in and opportunities for recreation/leisure, physical environment and transport). The children were required to answer all the questionnaire items except for the two asking for sexual activity and economical independence. However, the only domain considered for this study was the psychological area. The average score obtained from the items of the psychological area was multiplied by 4, in order to make it comparable with the score of the WHOQOL-100, and subsequently transformed into a scale from 0 to 100, using the following formula: (score − 4) × (100/16). The higher the score, the better the psychological adjustment.

##### Self-esteem

The Italian version of the Multidimensional Self Concept Scale (TMA according to the Italian name the test), was used to assess self-esteem^59,96–98^. The questionnaire examined the six following areas related to self-esteem: interpersonal area (social relationships with peers and adults); school area (successes and failures experimented in the classroom); emotional area (emotions and ability to regulate negative emotions); family area (relationships with family, feelings of love and value); body area (body aspect, physical and sports skills); sense of mastery of the environment area (one’s perceptions of the ability to control the environment). The questionnaire is composed of 150 items (25 for each area), each with 4 possible answers (absolutely true, true, not true, absolutely not true). Scores in each of the 6 self-esteem dimensions and a global self-esteem related score are generated. These scores were expressed as T-scores (M = 50, SD = 10), calculated on the basis of Italian normative data^59,98^. The higher the scores, the higher self-esteem levels.

### Covariates

We considered the following covariates

- Improvement on CCT tasks (practice-related improvement): The LPI index, which was automatically supplied by the Lumosity Cognitive Training™ web-platform, was used as a measure of improvement with respect to CCT tasks^56^. This index assessed the average level of performance across training games. This measure was age-adjusted, but not standardized. The improvement on training tasks was calculated as the difference in LPI between the last and the first day of training (LPI-change).
- FSIQ: Intellectual functioning was assessed at baseline evaluation through Wechsler Intelligence Scales Fourth Edition (WISC-IV)^99^. This scale provides a Verbal Comprehension Index (VCI), a Perceptual Reasoning Index (PRI), a Working Memory Index (WMI), a Processing Speed Index (PSI) and a FSIQ. FSIQ has an M of 100 and an SD of 15.

### Data handling and statistical analysis

Demographic, clinical and neuropsychological variables were described through descriptive statistics. A modified intention to treat analysis approach was used, including in the analysis all the participants that had undergone the pre- and post-treatment evaluation sessions, even if they did not complete all the CCT sessions; no imputation of missing data was used, considering the limited sample size and observation points. Preliminary analyses were conducted to detect potential differences in demographical, clinical, cognitive and psychological adjustment measures, assessed at T1, among diagnostic groups by means of a series of one-way ANOVAs. Furthermore, a series of independent t-tests were conducted to detect potential differences among the Training-first group and the Waiting-first group in the primary and secondary cognitive outcomes at T1.

In order to test the efficacy of the CCT in the Training-first group and in the Waiting-first group, we calculated the change between T1 and T2 (delta 1) and between T2 and T3 (delta 2), measuring the difference between the second and the first time point. Delta measures were entered into a series of 2 × 2 mixed ANOVAs performed to compare the change between T1 and T2 (delta 1) and between T2 and T3 (delta 2) in the two groups (Training-first vs. Waiting-first). Delta time was inserted as a within-subject variable and Group as a between-subject factor. Furthermore, as participants displayed heterogeneous levels of intellectual functioning, results were controlled for the possible influence of individual intellectual ability and of practice-related improvements on the trained tasks (LPI). Thus, whenever the main ANOVA showed significant interaction effects, the FSIQ at baseline and the change of LPI between the first and the last training session (LPI-change) were inserted into follow-up ANCOVAs as covariates. Furthermore, dependent-sample t-tests (one-tailed) were used to assess the long-lasting effects of the training, by comparing the 6-month-follow-up and the scores obtained in the pre-training session (i.e., T1 vs. T4 for the Training-first group and T2 vs. T5 for the Waiting-first group) of the measures showing a significant improvement after the training.

Finally, for those measures that resulted to be enhanced by the training, we run exploratory between-group comparisons using one-way factorial ANOVA to evaluate whether the treatment effects (i.e., delta 1 for the Training-first group and delta 2 for the Waiting-first group) were different across the three diagnostic groups (i.e., non-progressive ABI, BT, CBM).

Analyses were performed using STATISTICA 8.0.360 for Windows. Significance threshold was set at *p* < 0.05 for all tests. Post hoc analysis was computed using Duncan’s test. Effects sizes were reported as partial eta squared (η^2^_p_) for the ANOVA and as Cohen’s d for significant effects and the follow-up pairwise comparisons, and interpreted according to standard benchmarks.

## Data Availability

Data supporting the results is available upon reasonable request to be directed to the corresponding author (viola.oldrati@lanostrafamiglia.it).
